# Efgartigimod non-responders after the first treatment cycle in generalized myasthenia gravis: a retrospective analysis of predictive factors

**DOI:** 10.3389/fneur.2025.1715486

**Published:** 2025-11-06

**Authors:** Zhenyu Niu, Jianchun Wang, Jingru Ren, Ran Liu, Jing Guo, Nan Zhang, Yiming Zheng, Hongjun Hao, Feng Gao, Haiqiang Jin

**Affiliations:** 1 Department of Neurology, Peking University First Hospital, Beijing, China; 2 Rare Disease Medical Center, Peking University First Hospital, Beijing, China

**Keywords:** generalized myasthenia gravis, efgartigimod, treatment response, eculizumab, predictors, retrospective study

## Abstract

**Objective:**

This study aimed to identify predictors of suboptimal response to efgartigimod in patients with generalized myasthenia gravis (gMG).

**Methods:**

In this single-center retrospective study, 35 gMG patients treated with efgartigimod were categorized into responders (*n* = 25) and non-responders (*n* = 10). Responders were defined by a reduction of >2 points in MG-ADL or >3 points in QMG score after one cycle, whereas non-responders showed improvement below these thresholds and subsequently responded to eculizumab. Demographic, clinical, and serological features were compared using univariate and multivariate analyses.

**Results:**

Non-responders had higher baseline gross motor and respiratory sub-scores. Univariate analysis revealed that thymoma, non-thymoma tumors, thyroid disease, and other autoimmune diseases were more common in non-responders. Multivariate analysis indicated that combinations of these factors were associated with a high predicted probability of poor response (up to 97.9%).

**Interpretation:**

Comorbidities including thymoma, other tumors, thyroid disease, and additional autoimmune disorders may predict reduced response to efgartigimod in gMG patients. Systematic evaluation of these factors could help optimize treatment selection.

## 1 Introduction

Efgartigimod, a human Immunoglobulin(Ig) G1 Fc fragment, targets the neonatal Fc receptor (FcRn) to promote IgG degradation, thereby reducing IgG levels (1). It demonstrated efficacy and safety in the ADAPT trial and its extension, leading to its approval for generalized myasthenia gravis (gMG) (2, 3). Now included in gMG treatment guidelines, efgartigimod exhibits a rapid onset of action and selectively lowers IgG without affecting other immunoglobulins (4–6). Although initially studied in acetylcholine receptor antibody (AChR-Ab)-positive patients, real-world evidence supports its use in types with other antibodies (7). In contrast, eculizumab—a complement C5 inhibitor—is supported by the REGAIN trial and real-world studies for AChR-Ab-positive gMG refractory to conventional therapy, though its efficacy remains unestablished in other types (8, 9).

Not all gMG patients respond adequately to efgartigimod. Across several trials and cohort studies, response rates—as measured by improvements in Myasthenia Gravis Activities of Daily Living (MG-ADL) or Quantitative Myasthenia Gravis (QMG) scores—range between approximately 64 and 85% (10–12). One multicenter real-world study conducted in China reported a 97% response rate based on MG-ADL assessment, which stands as an outlier (13). In comparison, reported response rates for eculizumab range between 60 and 77% (8, 14), similar to those of efgartigimod. Suboptimal response to efgartigimod poses a clinical challenge, particularly due to its potential impact on other acute treatments such as intravenous immunoglobulin (IVIg), as well as an additional economic burden on patients. Previous studies have rarely characterized efgartigimod non-responders. One retrospective cluster analysis of gMG patients treated with efgartigimod suggested poorer responses in those with thymoma-associated myasthenia gravis (TAMG) (15), a finding supported by a cohort study of three non-responders (13). Another real-world study further indicated that 8 out of 9 TAMG patients who did not respond to efgartigimod showed significant improvement after switching to eculizumab (16). However, not all TAMG patients are non-responders; a phase II trial of perioperative efgartigimod in 40 TAMG patients reported clinical remission in all cases (17). Existing evidence remains limited regarding other predictive factors.

Therefore, this article presents a retrospective analysis of a cohort of gMG patients treated with efgartigimod at our center. Patients were grouped according to treatment response, and both univariate and multivariate analyses were conducted to identify predictors of efficacy. The aim is to contribute to treatment optimization and support the development of individualized immunotherapy strategies for gMG.

## 2 Methods

### 2.1 Study design and patients

This single-center, retrospective study consecutively screened and included all patients with a confirmed diagnosis of gMG who were hospitalized at Peking University First Hospital between January 2024 and March 2025 and received at least one full standardized treatment cycle of efgartigimod (one infusion per week for 4 weeks). Clinical data before and after the first treatment cycle were collected.

A good response to treatment was defined as either a reduction of >2 points in the MG-ADL score or a reduction of >3 points in the QMG score after 4 weeks of regular efgartigimod treatment, including patients who achieved clinical minimal manifestation (CMI) (2). Conversely, a poor response was defined as an improvement of <2 points in MG-ADL or <3 points in QMG scores. All outcome assessments were performed by the same dedicated panel of three qualified clinicians who had not taken part in the research in order to ensure consistency.

Patients demonstrating a good response were classified as responders. The non-responder group was specifically defined as those patients who not only had a poor initial response to efgartigimod (improvement of <2 points in MG-ADL or <3 points in QMG) but also subsequently demonstrated a favorable response after switching to eculizumab. Demographic factors, clinical characteristics, underlying diseases, and comorbidities were compared between these two groups. It is critical to note that only AChR-Ab positive patients with a poor response to efgartigimod were considered for switching to eculizumab, in accordance with its approved indication. Therefore, the non-responder group is exclusively composed of AChR-Ab positive patients.

All patients provided signed informed consent forms, and the study protocol was approved by the Institutional Review Board of Peking University First Hospital.

### 2.2 Clinical evaluation

All patients included in this study met the diagnostic criteria established by the internationally recognized 2022 Japanese Clinical Guidelines for Myasthenia Gravis (18). This required the presence of fluctuating skeletal muscle weakness and fatigability, along with the detection of pathogenic autoantibodies including AChR-Ab, muscle-specific tyrosine kinase antibody (MuSK-Ab), or lipoprotein-related protein 4 antibody (LRP4-Ab) and/or supportive evidence of impaired neuromuscular transmission. Confirmatory tests included a positive neostigmine test, a decremental response (>10%) in compound muscle action potentials (CMAP) on low-frequency repetitive nerve stimulation (RNS), or increased jitter on single-fiber electromyography (SFEMG). Pathogenic autoantibodies were detected using enzyme-linked immunosorbent assay (ELISA) with widely validated commercial kits. In addition to the three primary pathogenic autoantibodies, testing for acetylcholinesterase antibody (AChE-Ab), Titin antibody (Titin-Ab), and ryanodine receptor antibody (RyR-Ab) was also performed.

Following diagnosis and prior to initiating efgartigimod treatment, all patients underwent a comprehensive baseline assessment. This included evaluation of disease severity and activity, screening for thymic pathology via chest high-resolution computed tomography (HRCT), screening for other systemic malignancies and autoimmune diseases, pulmonary function tests, and diaphragmatic ultrasonography to assess regional muscle involvement. Disease severity was classified according to the Myasthenia Gravis Foundation of America (MGFA) clinical classification (19). Baseline disease severity and activity were further quantified using the MG-ADL scale, the QMG score (20), and the Myasthenia Gravis Composite (MGC) score (21), in accordance with international standards.

All enrolled patients received the standard regimen of efgartigimod, which consisted of a 10 mg/kg intravenous infusion administered once weekly for four consecutive weeks. Throughout the period from 2 months prior to initiating efgartigimod until 1 month after the administration of either efgartigimod or eculizumab, none of the patients received acute-phase treatments such as high-dose intravenous corticosteroid pulse therapy, IVIg, or plasma exchange. Additionally, no other biologic agents were administered. The use of oral corticosteroids and oral immunosuppressants was also documented and analyzed.

After the initiation of efgartigimod (defined as Week 0), disease severity scores (MG-ADL, QMG, and MGC) were reassessed at two timepoints: 1 week after the second infusion (Week 2) and 1 week after the fourth infusion (Week 4). Patients in the non-responder group switched to eculizumab shortly after completing the fourth efgartigimod infusion. They then received an initial dosing regimen of eculizumab (600 mg weekly for 4 weeks). These patients similarly underwent repeat MG-ADL, QMG, and MGC assessments 1 week after the second (Week 2) and 1 week after the fourth (Week 4) eculizumab infusions.

### 2.3 Statistical analysis

All statistical analyses were performed using the R programming language. As most continuous variables did not meet the assumptions of normality, the Mann–Whitney U test was used to assess differences between groups, with a *p*-value < 0.05 considered statistically significant. Given the small sample size, Fisher’s exact test was applied for comparing proportional differences between two groups, while the Kruskal-Wallis H test was used for comparisons across more than two groups. Additionally, the likelihood ratio test was employed to evaluate the collective effect of all factors on treatment response.

Finally, Firth’s penalized-likelihood regression was utilized for multivariable analysis involving two variables to mitigate bias due to limited sample size. Since all patients exhibited ocular muscle weakness and had a positive ocular neostigmine test—and the likelihood ratio test requires factors with two or more levels—these two invariant factors were excluded, leaving 21 factors for the overall assessment.

## 3 Results

### 3.1 Participants and grouping

A total of 35 patients with generalized myasthenia gravis (gMG) were included in this study. Based on treatment response after one cycle of efgartigimod, 25 patients were classified as responders and 10 as non-responders, the latter showing marked improvement after switching to eculizumab. Baseline characteristics are summarized in Table 1.

**Table 1:** General information and clinical features of patients.

Information	Responder	Non-responder	*p*-value
Number	25	10	\
Sexual			
Female	44.00% (11/25)	80.00% (8/10)	0.071
Male	56.00% (14/25)	20.00% (2/10)	
Age at Onset	63.00 (55.50 ~ 72.00)	60.00 (52.75 ~ 70.25)	0.483
Pre-treatment illness duration (month)	24.00 (3.00 ~ 72.00)	36.50 (6.75 ~ 73.50)	0.815
Pathogenic autoantibody			
AChR-Ab	92.00% (22/25)	100.00% (10/10)	1.000
MuSK-Ab	8.00% (2/25)	0	
Negative	4.00% (1/25)	0	
AChR-Ab Titer	1.60 (1.07–2.18)	1.69 (1.34–2.11)	0.575
Other autoantibody			
AChE-Ab	4.00% (1/25)	20.00% (2/10)	0.190
Titin-Ab	68.00% (17/25)	80.00% (8/10)	0.686
RyR-Ab	24.00% (6/25)	20.00% (2/10)	1.000
MGFA clinical classification			
IIa	48.00% (12/25)	40.00% (4/10)	0.843
IIb	24.00% (6/25)	40.00% (4/10)	
IIIa	4.00% (1/25)	0	
IIIb	16.00% (4/25)	10.00% (1/10)	
IVa	0	0	
IVb	4.00% (1/25)	10.00% (1/10)	
V	4.00% (1/25)	0	
Clinical features			
BMI	24.34 (21.45–27.08)	26.18 (24.22–27.36)	0.303
Smoking	20.00% (5/25)	30.00% (3/10)	0.661
Heavy drinking	16.00% (4/25)	10.00% (1/10)	1.000
Hypertension	68.00% (17/25)	50.00% (5/10)	0.444
Diabetes	40.00% (10/25)	40.00% (4/10)	1.000
Hyperlipidemia	52.00% (13/25)	50.00% (5/10)	1.000
Thyroid disease	4.00% (1/25)	30.00% (3/10)	0.061
Cardiovascular disease	20.00% (5/25)	20.00% (2/10)	1.000
Cerebrovascular disease	24.00% (6/25)	10.00% (1/10)	0.644
Pulmonary disease	20.00% (5/25)	0	0.292
Gastrointestinal disease	20.00% (5/25)	30.00% (3/10)	0.661
Other autoimmune disease	4.00% (1/25)	50.00% (5/10)	0.004**
Tumor (exclude thymoma)	4.00% (1/25)	40.00% (4/10)	0.017*
Myasthenic crisis history	40.00% (10/25)	10.00% (1/10)	0.089
Thymoma	16.00% (4/25)	40.00% (4/10)	0.140
Thymectomy	100.00% (4/4)	25.00% (1/4)	0.071
Ocular neostigmine test positive	100.00% (25/25)	100.00% (10/10)	\
RNS decremental response	72.00% (18/25)	80.00% (8/10)	1.000
FVC Decrease >10%	48.00% (12/25)	40.00% (4/10)	0.723
Diaphragm US abnormality	36.00% (9/25)	20.00% (1/10)	0.447

AchR-Ab, acetylcholine receptor antibody; MuSK-Ab, muscle-specific tyrosine kinase antibody; Titin-Ab, Titin antibody; RyR-Ab, ryanodine receptor antibody; MGFA, Myasthenia Gravis Foundation of America; RNS, repetitive nerve stimulation; FVC, forced vital capacity; US, ultrasonography.*Indicates *p* < 0.05.**Indicates *p* < 0.01.

Demographic analysis revealed that two groups were comparable in age (median 63 vs. 60 years, *p* = 0.483) and pre-treatment disease duration (*p* = 0.815). All non-responders were AChR-Ab positive, whereas the responder group included two MuSK-Ab positive and one seronegative patient. Titers of AChR-Ab were no significant differences between two groups (*p* = 0.575). There were no significant differences in the prevalence of other MG-related antibodies (AChE-Ab, Titin-Ab, RyR-Ab) or in MGFA clinical classifications (*p* = 0.843).

### 3.2 Treatment and therapeutic response of patients

All patients, except for three classified as MGFA type IV or above (two in the responder group and one in the non-responder group), received the maximum tolerated dose of oral pyridostigmine bromide. Details regarding other immunotherapy regimens are provided in Table 2. There were no significant differences between the two groups in the proportions of patients receiving oral corticosteroids, immunosuppressants, or combination therapy. Notably, all patients treated with oral corticosteroids successfully underwent corticosteroid dose reduction following treatment with efgartigimod or eculizumab.

**Table 2:** Other immunotherapy of patients.

Therapy	Responder	Non-responder	*p*-value
Efgartigimod only	24.00% (6/25)	30.00% (3/10)	0.694
Oral corticosteroids	20.00% (5/25)	20.00% (2/10)	1.000
Immunosuppressants			
Methotrexate	8.00% (2/25)	10.00% (1/10)	0.959
Mycophenolate mofetil	20.00% (5/25)	20.00% (2/10)	
Tarolimus	28.00% (7/25)	20.00% (2/10)	
Azathioprine	4.00% (1/25)	0	
Corticosteroids with immunosuppressants	8.00% (2/25)	0	1.000

**Figure 1:**
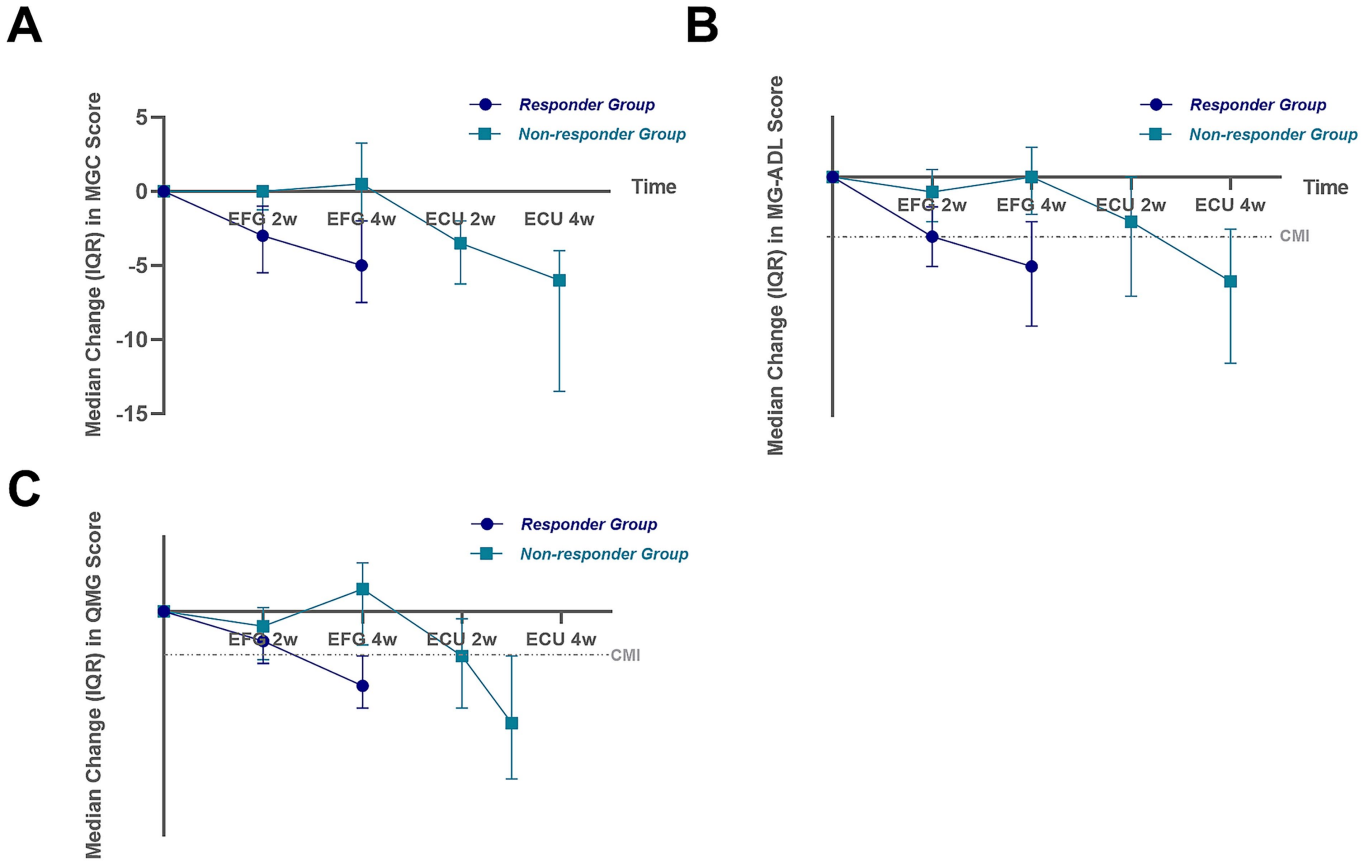
Clinical scores (MGC, MG-ADL and QMG) of two groups throughout the treatment. Changes from baseline of MGC score **(A)**, MG-ADL score **(B)** and QMG score **(C)** of two groups by time, evaluated at 1 week after the second (Week 2) infusion and 1 week after the fourth (Week 4) infusion. CMI, clinical minimal manifestation, defined as either a reduction of >2 points in the MG-ADL score or a reduction of >3 points in the QMG score.

Treatment response to efgartigimod significantly differed between the two groups (Figure 1). Responders showed early and sustained improvements: by week 4, median reductions from baseline were 5 points in MGC, 3 in MG-ADL, and 5 in QMG scores (all *p* < 0.001). The proportion achieving CMI reached 88.0% by MG-ADL and 96.0% by QMG criteria, with all responders meeting CMI in at least one scale.

In contrast, non-responders exhibited no significant improvement after efgartigimod treatment. At week 4, median changes in MG-ADL, MGC, and QMG scores were 0, +0.5, and +1.5, respectively (all *p* > 0.05), and no patient achieved CMI. After switching to eculizumab, all non-responders showed significant improvement: median reductions were 6 points in MGC, 3.5 in MG-ADL, and 7.5 in QMG (all *p* < 0.001), with all achieving CMI.

### 3.3 Comparison of pre-medication scores across groups

**Figure 2:**
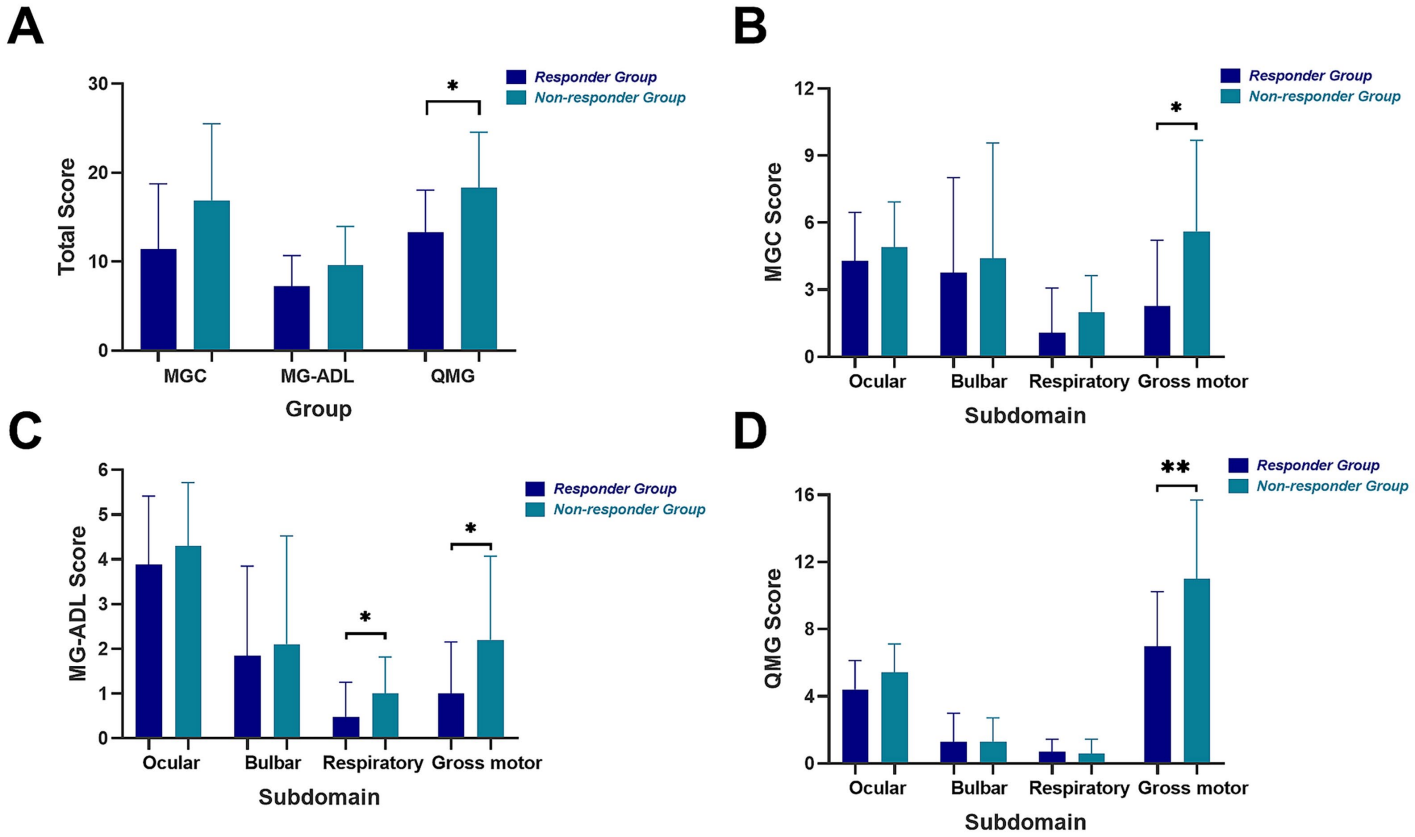
Total clinical scores and scores by subdomains of two groups. Total MGC, MG-ADL, QMG scores **(A)** and MGC score **(B)**, MG-ADL score **(C)** and QMG score **(D)** by four subdomains including ocular, bulbar, respiratory and gross motor of two groups. Ocular subdomain: diplopia, ptosis and eyelid closure strength. Bulbar subdomain: speech, voice, swallowing and chewing. Respiratory subdomain: breathing to activity and forced vital capacity measurement. Gross motor subdomain: limb and neck muscle function.

Following the confirmation of differential treatment responsiveness to efgartigimod between the two groups, we compared the overall baseline clinical scores and performed a detailed subdomain analysis for each score, stratified by affected muscle groups. Baseline clinical scores were generally higher in non-responders across MGC, MG-ADL, and QMG scales (Figure 2A), though only the difference in QMG scores reached statistical significance (median 20 vs. 13, *p* = 0.013). Subdomain analysis revealed that non-responders had significantly higher gross motor scores across all rating scales (MGC: *p* = 0.028; MG-ADL: *p* = 0.041; QMG: *p* = 0.004). Respiratory sub-scores were also elevated in non-responders on both MGC (*p* = 0.083) and MG-ADL (*p* = 0.043), though not on the QMG respiratory subdomain (*p* = 0.733) based on forced vital capacity (FVC) (Figures 2B–D). No significant differences were observed in ocular or bulbar sub-scores in any of the three scales.

### 3.4 Univariate analysis of clinical features on therapeutic response

Univariate analysis was performed to identify factors potentially influencing efgartigimod response (Table 1). No significant differences were found in lifestyle factors (long-term smoking, heavy drinking) or common chronic diseases (hypertension, diabetes, hyperlipidemia as well as cardiovascular, cerebrovascular, gastrointestinal and pulmonary diseases) between groups. Non-responders had a higher prevalence of thyroid disease (30.0% vs. 4.0%, *p* = 0.061), other autoimmune diseases excluding autoimmune thyroid diseases (50.0% vs. 4.0%, *p* = 0.004), and non-thymoma tumors (40.0% vs. 4.0%, *p* = 0.017). Thymoma was also more common in non-responders (40.0% vs. 16.0%, *p* = 0.140), and notably, only 1 of 4 non-responders with thymoma had undergone thymectomy, compared to 4/4 in the responder group. A history of myasthenic crisis was less frequent in non-responders (10.0% vs. 40.0%, *p* = 0.089). Detailed classifications, pathological types, and stages of these comorbidities (thymomas, other tumors, autoimmune diseases, and thyroid disorders) are provided in Supplementary Table S1.

### 3.5 Multivariate analysis of risk factors on low treatment effect

**Figure 3:**
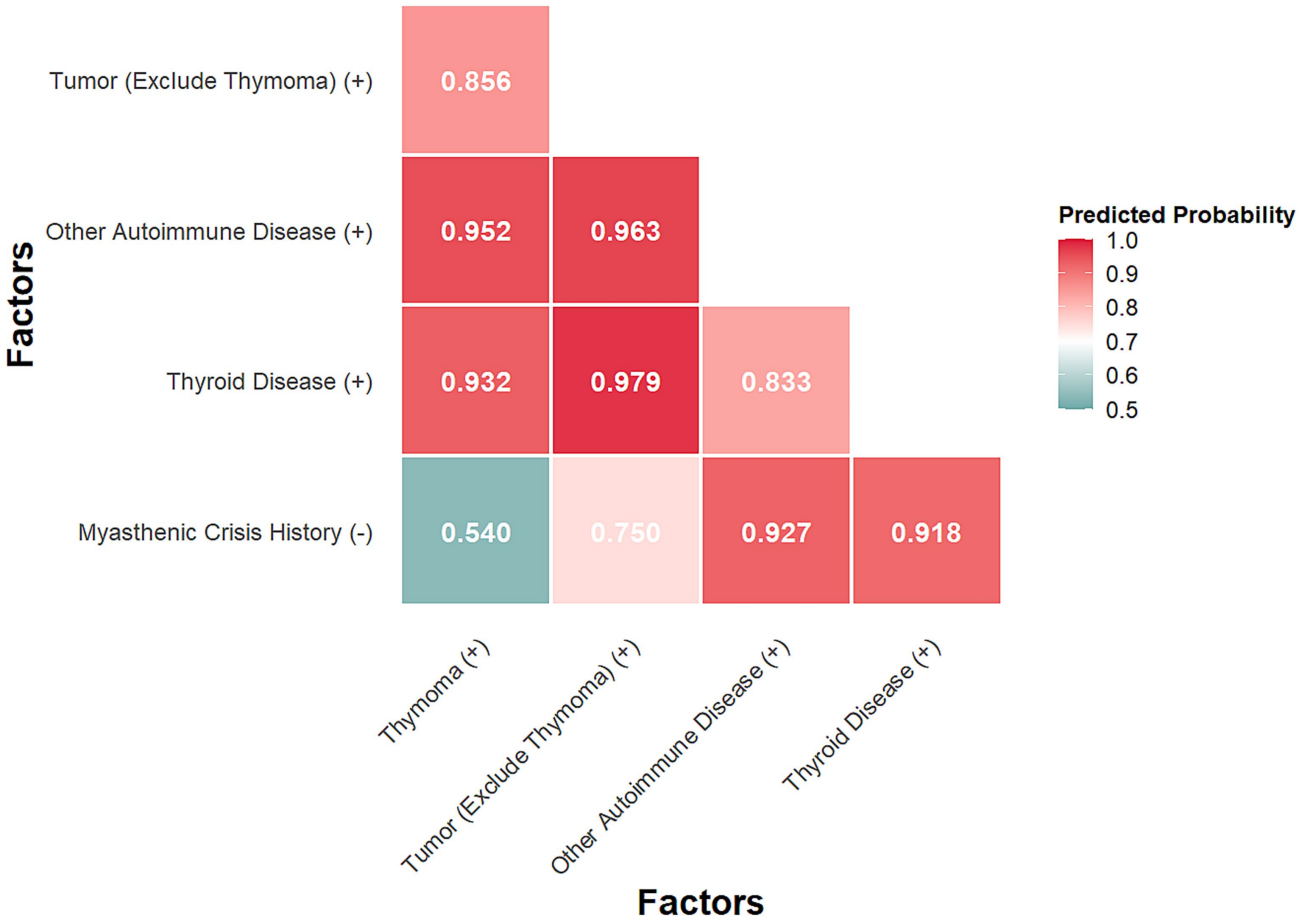
Predicted probability by Firth’s multivariate analysis.

The Likelihood Ratio Test indicated that these 21 clinical features were collectively significantly associated with treatment response (χ^2^ = 41.879, df = 21, *p* = 0.004). Furthermore, based on the univariate analysis of 18 clinical features, 5 factors were selected for further multivariate assessment using pre-specified criteria (*p*-value < 0.2 and 95% CI of OR excluding 1). These included: Thymoma, Tumor (excluding thymoma), Thyroid Disease, Other Autoimmune Disease, and History of Myasthenic Crisis. The first four were positively associated with non-response, while history of myasthenic crisis was negatively associated. All pairwise combinations of these factors significantly predicted treatment outcome (all *p* < 0.05; Supplementary Table S1). The predicted probabilities of poor response are shown in Figure 3. The combination of Tumor (excluding thymoma) and Thyroid Disease showed the highest predicted probability of poor response (0.979), followed by Tumor and Other Autoimmune Disease (0.963), and Other Autoimmune Disease with Thymoma (0.952). Combinations including no history of myasthenic crisis yielded the lowest probabilities, notably with Thymoma (0.540).

## 4 Discussion

In recent years, targeted biologic agents have been developed to overcome limitations of conventional gMG therapies, such as nonspecific side effects, delayed onset of action, and inadequate control of refractory or relapsing disease. As a downstream rescue therapy, efgartigimod has shown significant efficacy and a favorable safety profile in both clinical trials and real-world studies involving both AChR-Ab-positive and negative patients (2, 3, 7, 22). Alternatively, eculizumab provides another targeted option for AChR-Ab-positive patients, with some cohort studies reporting comparable or slightly higher improvement rates relative to efgartigimod (23). However, due to its relatively recent introduction into clinical practice, few studies have focused specifically on patients who respond poorly to efgartigimod. Thymoma has been proposed in a limited number of studies as a potential risk factor for suboptimal response to efgartigimod (15, 16)—and conversely, a predictor of better response to eculizumab—though some reports present conflicting conclusions. All patients in our defined non-responder group were AChR-Ab positive, which provided the clinical and pathophysiological basis for switching to eculizumab. This approach allowed us to identify a cohort of patients with a truly refractory, antibody-driven disease who, despite not responding to IgG reduction via FcRn blockade, showed marked improvement with complement inhibition, highlighting a distinct mechanism-specific treatment failure and subsequent success.

Demographic analysis revealed no significant difference in general information and other treatment, while three AChR-Ab negative patients in the responder group showed notably improvement, supporting existing evidence that efgartigimod benefits gMG patients regardless of antibody status. As per inclusion criteria, responders all achieved CMI after one cycle of efgartigimod, whereas non-responders showed no significant improvement—some even worsened. All non-responders subsequently achieved CMI after switching to eculizumab. Given that both biologics target antibody mediated pathways—efgartigimod enhancing IgG degradation and eculizumab inhibiting complement activation—the differential response supports the antibody-driven nature of disease in both groups and justifies our focus on eculizumab-responsive non-responders. Subdomain analysis indicated significantly higher gross motor and respiratory scores in non-responders at baseline, particularly in gross motor function across all clinical scales. The lack of significant difference in the respiratory subdomain of QMG may relate to its objective FVC-based assessment, potentially influenced by higher pulmonary comorbidity rates in responders, and limited sensitivity to fatigability.

Univariate analysis revealed no significant differences in lifestyle factors, common chronic diseases, clinical symptoms, or diagnostic results. However, non-responders showed significantly higher rates of thymoma, other tumors, thyroid disease, and additional autoimmune disorders. Notably, although thymoma was present in both groups, all responders had undergone thymectomy compared to only one non-responder, suggesting that unresected thymoma may impair treatment response. The underlying mechanisms for this observation may be twofold. As detailed in Supplementary Table S1, the thymomas in non-responders were predominantly of types B1 and B2, which are known to be associated with a more robust lymphocytic component and potentially greater CD4 + CD8 + double-positive T-cell activity (24), suggests that TAMG is driven by a more complex autoimmune response involving prominent and persistent T-cell mediated autoimmunity (25, 26), against which FcRn antagonism may have limited efficacy. Second, an unresected thymoma can function as a permanent reservoir for generating new autoreactive lymphocytes, potentially outpacing the rate of pathogenic IgG clearance by efgartigimod. The critical role of persistent tumor burden is underscored by our finding that thymectomy was universal in responders with thymoma but rare in their non-responder counterparts. Moreover, other findings indicate that broader immune dysregulation, particularly involving autoimmunity and neoplasia, may contribute to reduced efgartigimod efficacy.

Interestingly, despite longer disease duration and higher clinical scores, fewer non-responders had a history of myasthenic crisis—a counterintuitive finding warranting further study. We hypothesize that this counterintuitive finding might be explained by a distinct clinical phenotype in non-responders, characterized by chronic and fixed progression of gross motor and respiratory weakness rather than acute, fluctuating exacerbations typical of a myasthenic crisis. Alternatively, or concurrently, the higher comorbidity burden in these patients (e.g., tumors) might have led to more stringent medical surveillance, facilitating earlier intervention and crisis prevention.

Combinations involving thyroid disease, non-thymoma tumor, and other autoimmune diseases were associated with predicted probabilities of poor response exceeding 95%. The highest probability (97.9%) was observed for co-occurrence of thyroid disease and non-thymoma tumor. Given that a considerable proportion of thyroid disorders are autoimmune in origin, these findings underscore the importance of systematic screening for tumors and other autoimmune comorbidities in patients with gMG. This approach may inform the future development of comprehensive clinical models for the disease.

Major limitations include the small sample size and group imbalance, which restricted extensive modeling and increased vulnerability to sampling bias. Although we consecutively included all eligible patients, our stringent definition of non-responders, which required both an inadequate initial response to efgartigimod and a subsequent response to eculizumab, inherently introduces a selection bias. It excludes AChR-Ab positive patients who did not respond to either agent, as well as non-responders with other antibody types who were not eligible for eculizumab. Therefore, our predictors specifically identify patients who are non-responsive to efgartigimod but responsive to eculizumab. Clinical assessments were also incomplete due to practical constraints, omitting dynamic immunologic biomarkers. The retrospective nature of the study resulted in potential missing data points and variation in each patient’s time frame to final documented visit. In addition, this was a single center experience in North China. The proportion of patients receiving concomitant oral corticosteroids in our cohort (20%) is relatively low compared to some other real-world studies. This may reflect our institutional preference to minimize chronic steroid use, especially in patients with comorbidities like diabetes or tumors, when initiating novel biologic agents (4, 7, 11, 12).

This retrospective analysis identifies specific comorbidities—including thymoma, other tumors, thyroid disease, and additional autoimmune disorders—as potential predictors of suboptimal response to efgartigimod in patients with gMG. The presence of these factors, particularly in combination, was associated with a high probability of treatment failure, while prior thymectomy appeared to mitigate non-response. These findings highlight the role of systemic immune dysregulation in modulating therapeutic outcomes and support comprehensive screening for coexisting autoimmune and neoplastic conditions in gMG patients considered for efgartigimod therapy. Future large-scale studies are needed to validate these associations and develop predictive models for individualized therapy selection in gMG.

## Data Availability

The original contributions presented in the study are included in the article/Supplementary material, further inquiries can be directed to the corresponding authors.
