# Supplementary material for: Efgartigimod non-responders after the first treatment cycle in generalized myasthenia gravis: a retrospective analysis of predictive factors
Source: Front Neurol. 2025 Nov 6;16:1715486. doi: 10.3389/fneur.2025.1715486 (PMC12631618; doi:10.3389/fneur.2025.1715486)
Supplement: Supplementary file 1 [file Table_1.docx]

**Supplemental Table S1** **Detailed Clinical Characteristics of Comorbidities in Responder and Non-responder Groups**

| **Condition** | **Patient** | **Details** |
| --- | --- | --- |
| Thymoma | NR-03 | Thymoma AB |
|  | NR-04 | Thymoma mixed B1 and B2, with thymectomy |
|  | NR-06 | Thymoma B1 |
|  | NR-10 | Thymoma B2 |
|  | R-07 | Thymoma B2, with thymectomy |
|  | R-08 | Thymoma B1 and B2 predominantly with a minor component of B3, with thymectomy |
|  | R-19 | Thymoma A2, with thymectomy |
|  | R-21 | Thymoma A3 |
| Non-thymoma Tumors | NR-04 | Clear Cell Renal Cell Carcinoma Fuhrman 2, with resection |
|  | NR-05 | Lung adenocarcinoma T1bN0M0 and Bilateral benign neoplasm of ovaries, with resection |
|  | NR-08 | Breast Cancer T3N2M0, with resection and radiotherapy |
|  | NR-09 | Breast Cancer T0N1miM0, with resection |
|  | R-18 | Lung adenocarcinoma T2aN1M0, with resection |
| Other Autoimmune Disease | NR-01 | Rheumatoid arthritis |
|  | NR-02 | Type 1 diabetes mellitus |
|  | NR-06 | Ankylosing spondylitis |
|  | NR-07 | Rheumatoid arthritis |
|  | NR-09 | IgA nephropathy |
|  | R-24 | Systemic lupus erythematosus |
| Thyroid Disease | NR-01 | Graves’ disease |
|  | NR-02 | Hashimoto's thyroiditis |
|  | NR-07 | Hashimoto's thyroiditis |
|  | R-10 | Hashimoto's thyroiditis |
